# Preliminary Characterization of Voltage-Activated Whole-Cell Currents in Developing Human Vestibular Hair Cells and Calyx Afferent Terminals

**DOI:** 10.1007/s10162-014-0471-y

**Published:** 2014-06-19

**Authors:** Rebecca Lim, Hannah R. Drury, Aaron J. Camp, Melissa A. Tadros, Robert J. Callister, Alan M. Brichta

**Affiliations:** 1 The School of Biomedical Sciences and Pharmacy, Faculty of Health and Medicine, Hunter Medical Research Institute, The University of Newcastle, Callaghan, NSW 2308 Australia; 2 Biomedical Science, School of Medical Sciences, The University of Sydney, Sydney, 2006 Australia

**Keywords:** electrophysiology, human, hair cells, vestibular, development

## Abstract

We present preliminary functional data from human vestibular hair cells and primary afferent calyx terminals during fetal development. Whole-cell recordings were obtained from hair cells or calyx terminals in semi-intact cristae prepared from human fetuses aged between 11 and 18 weeks gestation (WG). During early fetal development (11–14 WG), hair cells expressed whole-cell conductances that were qualitatively similar but quantitatively smaller than those observed previously in mature rodent type II hair cells. As development progressed (15–18 WG), peak outward conductances increased in putative type II hair cells but did not reach amplitudes observed in adult human hair cells. Type I hair cells express a specific low-voltage activating conductance, *G*_K,L_. A similar current was first observed at 15 WG but remained relatively small, even at 18 WG. The presence of a “collapsing” tail current indicates a maturing type I hair cell phenotype and suggests the presence of a surrounding calyx afferent terminal. We were also able to record from calyx afferent terminals in 15–18 WG cristae. In voltage clamp, these terminals exhibited fast inactivating inward as well as slower outward conductances, and in current clamp, discharged a single action potential during depolarizing steps. Together, these data suggest the major functional characteristics of type I and type II hair cells and calyx terminals are present by 18 WG. Our study also describes a new preparation for the functional investigation of key events that occur during maturation of human vestibular organs.

## INTRODUCTION

Most of our understanding about the cellular development of human peripheral vestibular organs comes from anatomical studies, which have documented their early growth and maturation (Sans and Dechesne 1985, 1987). Anatomical differentiation of human vestibular hair cells and supporting cells begins at the end of the embryonic period when fetal crown-rump length (CRL) is between 16.5 and 26 mm or approximately 8–9 weeks gestation (WG, Dechesne et al. 1987). At this stage, hair cells have short, polarized hair bundles and exhibit anatomical features, including synaptic bodies, which are consistent with synapse development (Sans and Dechesne 1985). Concomitantly, invading primary afferent fibers are juxtaposed with these nascent hair cells and exhibit postsynaptic densities (Sans and Dechesne 1985). Therefore, innervation by primary afferent fibers precedes full hair cell differentiation. However, synapse development occurs in tandem with morphological differentiation as reported in mouse vestibular organs (Rüsch et al. 1998) and mouse cochlea (Mbiene et al. 1988). Limited anatomical data are available for human tissue beyond this time.

We know that in amniotes (reptiles, birds, and mammals), further anatomical and physiological differentiation results in the emergence of type I and type II hair cells. In mature vestibular epithelia, these hair cell types can be distinguished by three features: (1) shape, (2) their primary afferent contacts, and (3) whole-cell conductances. The ubiquitous type II hair cell, which is present in all vertebrates, is cylindrically shaped, contacted by conventional bouton-like afferent terminals, and expresses an assortment of voltage- and ligand-gated conductances including: outward and inward rectifiers, A-like conductances, and calcium activated K^+^ conductances. Type I hair cells, in contrast, have a constricted neck, are contacted by an enveloping “cup-like” or calyx afferent terminal, and express a unique low-voltage activated K^+^ conductance called *G*_K,L_. In rodents, the emergence of *G*_K,L_ marks the physiological differentiation of type I hair cells (Rüsch et al. 1998; Geleoc et al. 2004).

Although we know that mature human vestibular hair cells express type I and type II specific whole-cell conductances (Oghalai et al. 1998) similar to those seen in rodents, we have no data on the physiological differentiation of human hair cells. Even basic information about when each hair cell type begins to express characteristic whole-cell conductances is not known. From a broader perspective it means we do not know how data from rodents with rapid fetal development measured in days and weeks, translates to the relatively long human gestational period, measured in months. To address these significant shortcomings, we have established a semi-intact preparation of fetal human vestibular organs and have begun to examine the spatiotemporal expression of whole-cell conductances in hair cells and associated calyx primary afferent terminals during an important phase (11–18 WG) of peripheral vestibular development.

## MATERIALS AND METHODS

The University of Newcastle Human Ethics Committee approved all procedures. Written consent was obtained from all tissue donors. Apart from gestational age, no other identifying information was supplied. Gestational age was determined by three criteria: (1) the date of the last menstrual period, (2) ultrasound measurement of CRL, and (3) foot length. Tissue was obtained from electively terminated fetal material. There were no known instances of abnormalities. Tissue specimens were collected in cold glycerol-based artificial cerebrospinal fluid (ACSF; see below) and transported to The University of Newcastle. Time between the tissue collection and the dissection of epithelium was less than 1 h.

### Electrophysiological Experiments

**FIG. 1:**
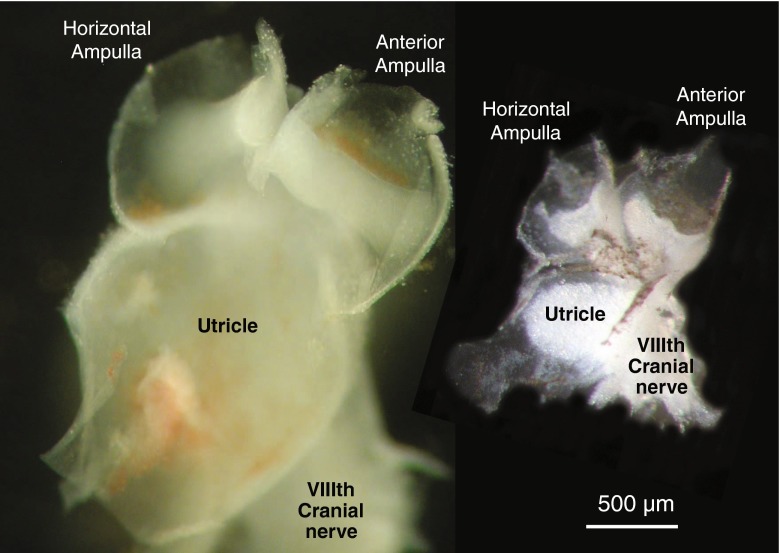
Isolated, semi-intact, inner ear preparations of left side peripheral vestibular organs from human fetus (13 WG) and mature mouse (3 weeks). The human (*left*) and mouse (*right*) neuroepithelial preparations consist of a horizontal and anterior ampullae and their associated cristae joined to the utricle forming a vestibular “triad,” together with remnants of the VIIIth cranial nerve. Membranous tissue and overlying accessory structures (cupulae and otoconial membranes) have been removed for direct visualization of epithelial surfaces. These preparations allow access to hair cells and afferent terminals for electrophysiological recordings. Even at this stage of development, human fetal vestibular organs are approximately twice the size of mature mouse vestibular organs. There are also a significantly greater number of hair cells and afferent fibers in human organs compared to mouse (see “DISCUSSION”).

Cold glycerol-based ACSF was used for tissue collection and transport (composition in mM: 250 glycerol, 26 NaHCO_3_, 11 glucose, 2.5 KCl, 1.2 NaH_2_PO_4_, 1.2 MgCl_2_, and 2.5 CaCl_2_). Glycerol-based ACSF has been used to improve cell viability by substituting glycerol for Na^+^. In central nervous system neurons, this reduces neurotoxicity, cell swelling, and lysis (Ye et al. 2006). The inner ears were isolated from the collected tissue and placed in a dissecting chamber filled with ice-cold glycerol-based ACSF and carbogenated with 5 % of CO_2_ and 95 % of O_2_. Throughout the developmental period investigated, the “bony” labyrinth of the inner ear was still cartilaginous and could be peeled away to expose the underlying membranous labyrinth. Compared to mature rodents, human fetal membranous labyrinths are larger, and at the ages examined, the individual ampullae are relatively less enlarged regions of the membranous ducts and the cristae are situated in closer proximity to the utricle. The membranous ducts were trimmed to reveal the utricle and anterior and horizontal cristae (Fig. 1). Together, these organs constituted our semi-intact neuroepithelial preparation. The preparation was transferred to a recording chamber containing oxygenated Liebovitz’s L15 cell culture medium (containing in mM: 1.26 CaCl_2_, 0.98 MgCl_2_, 0.81 MgSO_4_, 5.33 KCl, 0.44 KH_2_PO_4_, 137.93 NaCl, 1.34 Na_2_HPO_4_; Life Technologies, Australia; pH 7.45, 305 mOsM) and perfused at a rate of two bath volumes per minute (i.e., 3 ml/min).

Recording began approximately 90 to 120 min after elective termination, and the preparation remained viable for up to 5 h. Whole-cell recordings were made using borosilicate glass pipettes (3–5 MΩ) filled with potassium fluoride-based internal solution containing (in mM): 110 KF, 15 KCl, 27 KOH, 1 NaCl, 10 HEPES, 10 EGTA, 1.8 MgCl_2_, 3 d-glucose, 2 Na-ATP; pH 7.4 (Rennie and Streeter 2006; Lim et al. 2011), and all experiments were made at room temperature (22–25 °C). Liquid junction potential was calculated as ∼9 mV using JPCalc (Barry 1994), and data were corrected subtracting 9 mV from all potentials. Cells were identified with infrared differential interference contrast (IR-DIC) optics. In some cases, the fluorophore, Alexa-594 (0.2 %; Lim et al. 2011), was added to the internal solution to confirm cell morphology using an infrared CCD camera (DAGE-MTI, Michigan, USA) and Cool LED Precise Excite (LED wavelength 595 nm and ex 590/em 665 nm filter set; Chroma Technology, Vermont, USA). Recordings were obtained using an Axopatch 1D amplifier running Axograph X software and sampled at 20 kHz and filtered at 2–10 kHz. Series resistance was monitored during each recording session, and data were rejected if this changed by >20 %. Usually, we could record from more than one hair cell and/or calyx terminal in each sample. Only data from cristae are presented here. Instantaneous tail current–voltage (I–V) relationship were determined from voltage steps (−129 to +11 mV) to −39 mV. These activation curves were then fit using a Boltzmann equation to calculate *G*_MAX_, the maximum conductance; *V*_½_, potential at half-activation; and *S*, voltage required for an *e*-fold change in conductance (Lim et al. 2011). Series resistance compensation (60 %) was applied during recordings and compensated values are shown in Table 1. These recordings form a preliminary characterization of developing human fetal hair cells; therefore, no pharmacological agents were used in this investigation. Data are presented as mean ± SEM and were analyzed using Student’s *t* tests with appropriate tests for normality. “*n*” refers to the number of recorded cells.

**TABLE 1:** Properties and characteristics of type I and II vestibular hair cells aged 11–14 WG and 15–18 WG

	Type II		Type I
	11–14 WG	15–18 WG	15–18 WG
	*n* = 13	*n* = 17	*n* = 13
Ri (MΩ)	821.0 ± 150.0*	776.6 ± 139.3^#^	158.1 ± 37.4*^#^
Cm (pF)	12.0 ± 1.0	12.3 ± 1.2	13.03 ± 34.0
Rs (MΩ)	11.5 ± 4.0	7.7 ± 1.6	7.3 ± 2.4
*G*_max_ (nS)	3.5 ± 0.2*	11.9 ± 1.5*	
*V*_½_ (mV)	−22.4 ± 2.6	−25.6 ± 1.9	
Slope (mV)	7.9 ± 0.7	7.2 ± 0.4	

Values are mean ± SE. Specific *t* values are provided in the text

*^#^ *p* < 0.01, significant differences

## RESULTS

Whole-cell patch clamp recordings were obtained from hair cells and calyx afferent terminals in 31 semi-intact preparations of human fetal vestibular cristae (aged 11–18 WG).

### Hair Cell Recordings

**FIG. 2:**
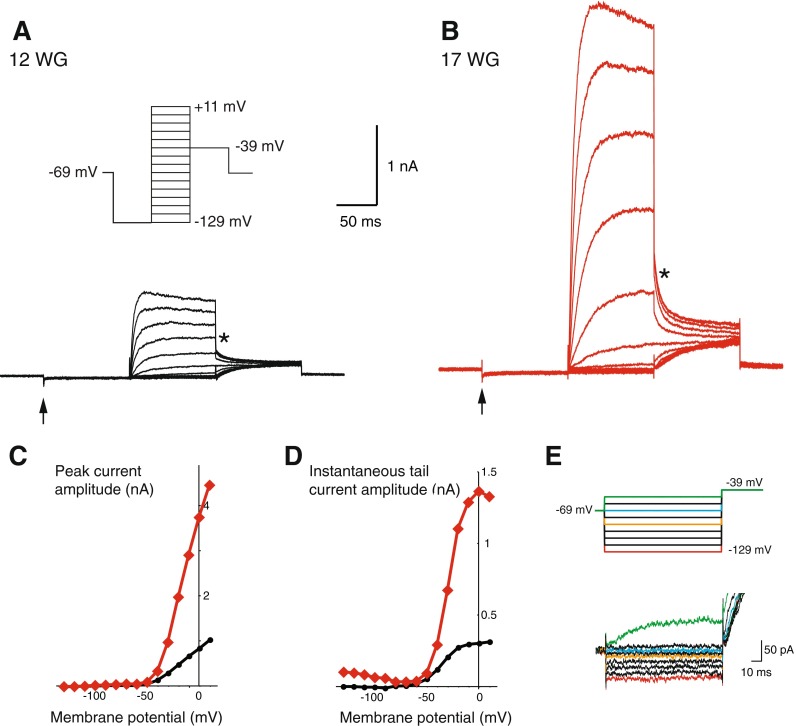
Whole-cell conductances in type II vestibular hair cells. A standard activation protocol (inset **A**) was used to generate whole-cell currents in putative type II hair cells. **A** Shows whole-cell conductances of an immature type II hair cell (12 WG). The *arrow* indicates when the cell is hyperpolarized to −129 mV. In this cell, there is little or no evidence for an inward rectifier or putative *G*_K,L_ conductance. **B** Shows the whole-cell conductances from a type II hair cell aged 17 WG are much larger than those from the hair cell in **A. C** Illustrates the I–V plot of peak conductance for type II hair cell aged 12 (*black trace*) and 17 (*red trace*) WG. **D** Activation curves were generated by plotting the extrapolated tail current values at the instant a −39 mV step was applied and plotting those values against the holding potentials, prior to the −39 mV step. Type II hair cells at both ages have sigmoidal activation curves that were fitted by Boltzmann equations. Calculated *G*_MAX_ value was larger in the type II hair cell aged 17 WG compared to the type II hair cell 12 WG. **E** In response to 100 ms voltage steps between −129 mV and −49 mV (*upper panel*), a type II hair cell aged 16 WG shows small inward currents at membrane potentials more negative than −89 mV (*orange*; *lower panel*).

We examined voltage-activated whole-cell currents in fetal vestibular hair cells. Recordings were made from a total of *n* = 51 hair cells. Figure 2 shows two presumptive type II hair cells, aged 12 and 17 WG, and their responses to a voltage protocol (2B inset) that first hyperpolarizes the cell with a −129 mV prepulse. In mature rodent hair cells, a hyperpolarizing prepulse distinguishes type I hair cells by deactivating a characteristic low-voltage conductance, *G*_K,L_ (Rüsch et al. 1998). Figure 2A, B shows neither cell exhibits a *G*_K,L_ conductance upon hyperpolarization, (i.e., no significant downward deflection at the arrowheads). Another feature of type II hair cells, compared to type I hair cells, is their relatively high input resistance. Both hair cells had high input resistances (826 and 1,587 MΩ, respectively). Group data shows that developing human hair cells without discernable *G*_K,L_ conductances have a high input resistance that is not significantly different between the two age groups (11–14 WG: 821.9 ± 150.0 MΩ, *n* = 13 versus 15–18 WG: 776.6 ± 139.3 MΩ, *n* = 17, *t* (28) = 2.05, *p* = 0.83; see Table 1).

Hair cells lacking *G*_K,L_ conductance exhibit an outward delayed rectifier conductance, G_DR_, which is similar to that expressed in type II hair cells throughout embryonic and postnatal development in rodent utricle and crista (Rüsch et al. 1998; Geleoc et al. 2004). The peak current–voltage (I–V) relationship of putative human type II hair cells increases with fetal age. For example, the 12 WG type II hair cell had a maximum peak current at +11 mV of ∼1 nA, which was substantially smaller than the peak current of ∼4.5 nA observed in the 17 WG type II hair cell (Fig. 2C). Similarly, group data shows that the peak current at +11 mV was significantly smaller at 11–14 WG than at 15–18 WG (1.0 ± 0.1 nA; *n* = 13 versus 2.5 ± 0.2 nA; *n* = 17, *t* (28) = 2.05, *p* = 0.00003).

Activation curves were obtained from the instantaneous tail currents immediately following a step to −39 mV (asterisks, Fig. 2A, B). These values were plotted against the holding potential prior to the step (Fig. 2D). Putative type II hair cells exhibit typical sigmoidal tail current activation curves. Boltzmann curves were fitted to calculate *G*_MAX_, *V*_½_, and *S* values. For the 12 WG type II hair cell, *G*_MAX_ was calculated as 3.9 nS versus 24.8 nS for the 17 WG type II hair cell. For group data, *G*_MAX_ was significantly smaller in putative type II hair cells aged 11–14 WG (3.5 ± 0.3 nS, *n* = 13) compared to those aged 15–18 WG (11.9 ± 1.5 nS, *n* = 17, *t* (28) = 2.05, *p* = 0.00008). Notably, even at this later developmental stage, hair cells exhibit only ∼60 % of the *G*_MAX_ values obtained in isolated adult human hair cells (11. 9 nS, *n* = 17 versus 19.3 nS calculated from Oghalai et al. 1998). These data support the notion that *G*_MAX_ values continue to increase with gestational age.

Although *G*_MAX_ values were smaller, *V*_½_ and *S* values were not significantly different (*t* (28) = 2.05, *p* = 0.32 and *t* (28) = 2.05, *p* = 0.43, respectively) between the two age groups (see Table 1). Furthermore, our data show that the calculated *V*_½_ and *S* values were similar to those obtained for embryonic mouse hair cells (Geleoc et al. 2004) and for *G*_DR1_ in postnatal rat hair cells (Rüsch et al. 1998). However, compared to isolated adult human hair cells (Oghalai et al. 1998), type II human fetal hair cells have a more depolarized *V*_½_ and larger *S* values or a less steep Boltzmann slope (human adult–estimated *V*_½_ = −47 mV, *S* = 6.1; human fetus *V*_½_ = −34.5 mV, *S* = 7.9).

In addition to outward conductances, at hyperpolarized membrane potentials (more negative than −70 mV), we observed small inward rectifying conductances (∼2 nS) at all age groups examined. Examples of limited inward rectification can be observed in Figure 2. However, there was no significant difference in the conductance of inward rectifying conductances between the two age groups (2.00 nS, *n* = 13 versus 1.73 nS, *n* = 17 for 11–14 WG and 15–18 WG, respectively; *t* (28) = 2.05, *p* = 0.64).

**FIG. 3:**
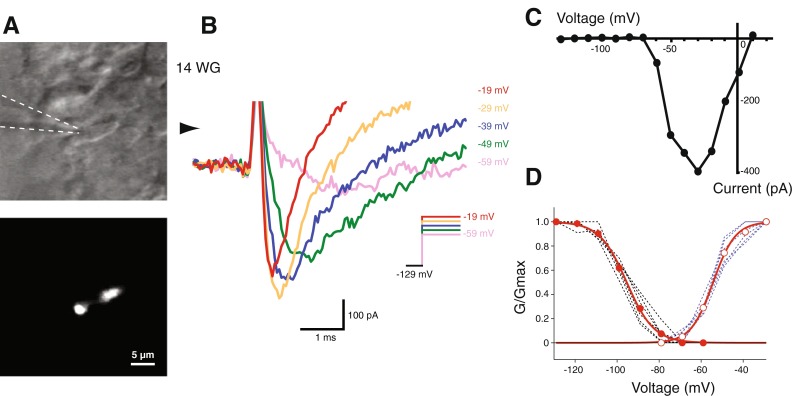
Fast inactivating inward currents in fetal vestibular hair cells. **A** Infrared DIC (*top*) and fluorescent image (*bottom*) shows a 14 WG vestibular hair cell. *Dashed lines* indicate recording electrode position. **B** Fast inactivating inward current in response to depolarizing voltage steps (shown in inset). *Arrowhead* indicates zero current. **C** I–V plot of the fast inactivating inward current resembles Na^+^ currents seen in developing rodent type I hair cells. **D** Activation (*blue*) and deactivation (*black*) and their respective average curves (*red*) of presumptive Na^+^ currents of immature hair cells. The average activation and deactivation curves have been fit with the Boltzmann equation, *V*_1/2 act_ = −54.3 mV, *S* = 5.3, and *V*_1/2 deact_ = −95.4 mV, *S* = −6.5, *n* = 6.

During early rodent embryonic development, some vestibular hair cells express fast-inactivating inward sodium (Na^+^) conductances (Wooltorton et al. 2007; Li et al. 2010). We observed similar fast-inactivating inward conductances in a subset of human vestibular hair cells but only up to ages of 14 WG. Figure 3A shows an IR-DIC image of a hair cell that had a high input resistance (890 MΩ) and did not exhibit a *G*_K,L_ conductance. Figure 3B shows the activation of inward currents in response to depolarizing voltage steps from −129 mV. In this cell, the inward current resembles a Na^+^ current and is activated at ∼−60 mV, with an apparent peak current amplitude of ∼400 pA at −29 mV (Fig. 3C). The magnitude of these inward currents is similar to those recorded from mouse utricular hair cells at E16 (Geleoc et al. 2004) and rat utricular hair cells at P1 (Wooltorton et al. 2007). In our sample, 8 of 38 hair cells that lacked the *G*_K,L_ conductance and had high input resistance (i.e., putative type II hair cells) displayed these inward conductances. These inward conductances were very fast, kinetically, and distinct from both slower inward rectifying conductances and larger outward conductances. We measured the putative Na^+^ currents in hair cells during activation and deactivation protocols and fit the data with Boltzmann functions (Fig. 3D). We did not attempt to pharmacologically isolate and identify these fast activating/deactivating currents in this initial characterization study of human fetal hair cells.

In addition to the type II hair cells described above, we made recordings from a total of 13 presumed type I hair cells. These were classified as type I hair cells on the basis of their low input resistance (158.1 ± 37.4 MΩ, *n* = 13) and the presence of a small putative *G*_K,L_ conductance. Although low input resistance is characteristically associated with type I hair cells (due to the presence of activated *G*_K,L_ at resting membrane potentials), this may also reflect a “leaky” cell. Therefore, to satisfy the type I designation, the hair cell had to show evidence of deactivation at more hyperpolarized potentials. In other words, membrane potentials more negative than -90 mV, where *G*_K,L_ is in a closed state, the hair cell’s input resistance was higher than at -69 mV when *G*_K,L_ is open (158 MΩ at −69 mV versus 306 MΩ at −99 mV, *n* = 5). The input resistance values obtained for these presumptive type I hair cells at −69 mV are comparable to that reported for embryonic mouse hair cells (158 versus 55 MΩ, Geleoc et al. 2004). The difference in input resistance between the hair cell types was not related to hair cell size, as measurements of membrane capacitance were similar in all hair cells (see Table 1).

**FIG. 4:**
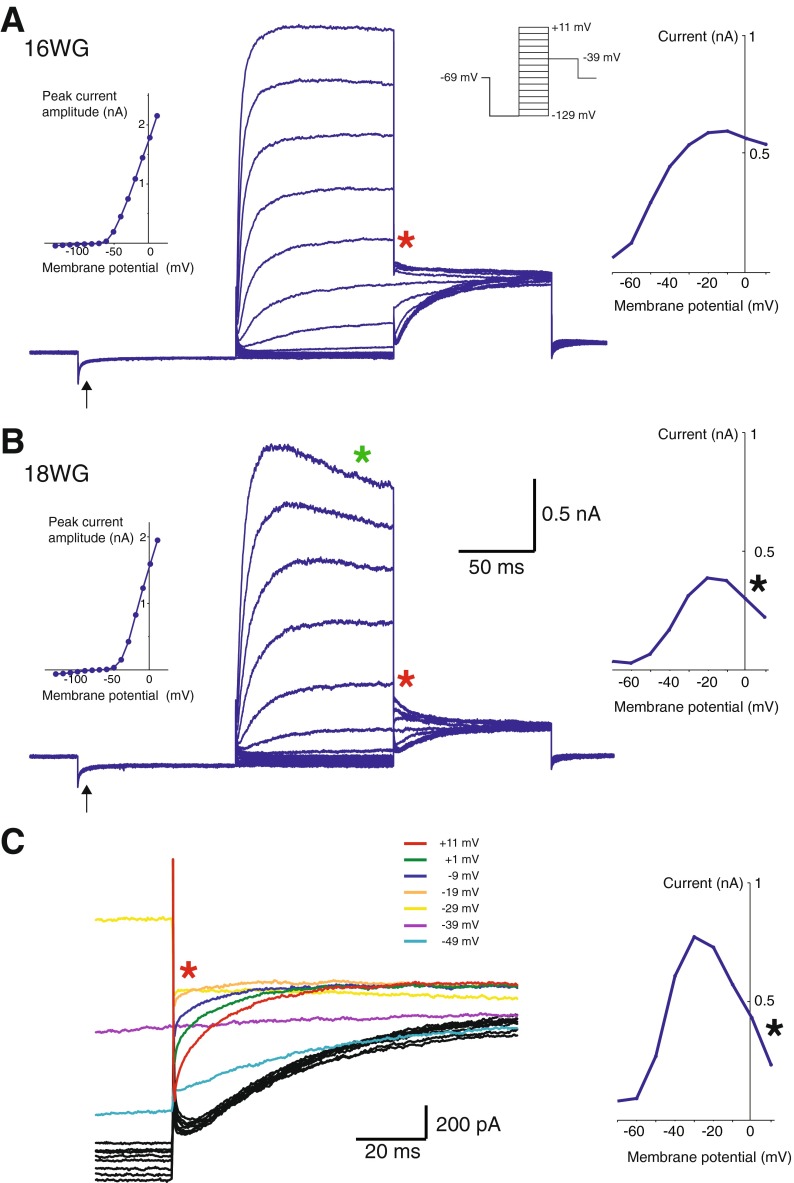
Whole-cell conductances from putative type I hair cells. **A** Shows whole-cell conductances from a putative type I hair cell in response to the voltage protocol (inset; *right*). Upon hyperpolarization to −129 mV, a presumed small *G*_K,L_ conductance is evident (*arrow*). This type I hair cell had fast onset and non-inactivating whole-cell conductances. A peak current I–V plot was generated for this cell (inset; *left*). Instantaneous tail currents (*red asterisk*) were used to produce an activation curve (inset; *right*). A Boltzmann equation was used to calculate *G*_MAX_, *V*_½_, and *S* values for this cell and were 11.2 nS, −47.4 mV, and 9.7, respectively. **B** Whole-cell conductances from another putative type I hair cell also shows the presence of a small *G*_K,L_ conductance (*arrow*) upon hyperpolarization to −129 mV. This cell displayed a small decline or “droop” (*green asterisk*) in current amplitude during steady-state activation. The inset (*left*) shows the peak current I–V plot for this cell. The activation curve (inset; *right*) produced from instantaneous tail currents (*red asterisk*) show a distinct “collapse” of the activation curve (*black asterisk*). In this example, *G*_MAX_, *V*_½_, and *S* values could not be calculated for this cell (see “DISCUSSION”). **C** Tail currents from a type I hair cell on an expanded time scale shows activation begins at ∼−49 mV and with a maximum peak current at −29 mV. At potentials more depolarized than −29 mV, the tail current reverses direction and “collapses” (*black asterisk*), a feature observed in embedded type I hair cells (Lim et al. 2011). The activation curve reflects this collapse and thus prevents accurate calculation of *G*_MAX_, *V*_½_, and slope.

Figure 4 shows two examples (A and B) of type I hair cells responding to the same voltage protocol as described for type II hair cells in Figure 2. In response to a hyperpolarizing voltage step to −129 mV, a small putative *G*_K,L_ conductance is evident (arrow). As described above for Figure 2, I–V plots of peak currents are shown as insets (left) in Figure 4A and B. These hair cells had a peak current at +11 mV of ∼2 nA which is smaller than the peak current of the type II hair cell shown in Figure 2B (∼4 nA) that is at a similar stage of development. Activation curves (insets; right) were plotted for these type I hair cells; however, at approximately −30 mV, the instantaneous tail currents reached a peak and then began to decrease or “collapse” (Fig. 4C).

We recently reported similar collapsing tail currents in type I hair cells from a semi-intact preparation of the mouse crista. This feature is not present in acutely *isolated* hair cells (Lim et al. 2011). In mice, we attributed the collapse of tail currents to the close apposition of the calyx terminal. These cup-like terminals surround type I hair cells early in fetal development (Sans et al. 1994) and restrict potassium (K^+^) diffusion away from the type I hair cell. This results in K^+^ accumulation between hair cell and calyx thereby reducing the driving force and attenuating tail currents (Lim et al. 2011). The collapsing tail currents in the putative type I hair cell at 15 WG suggests a similar situation exists in the human fetal hair cells; i.e., the presence of a developing partial or full calyx is sufficient to influence the ionic microenvironment around the type I hair cell. Importantly, while putative *G*_K,L_ is smaller in semi-intact human fetal type I hair cells, the presence of “collapsing” activation curves at 15 WG is indicative of an emerging type I hair cell.

As a consequence of collapsing activation curves, calculations of *G*_MAX_, *V*_½_, and *S* (which assume stable K^+^ concentrations surrounding the hair cell and fixed K^+^ reversal potentials) are not valid when analyzing type I hair cells and are therefore not presented.

### Calyx Observations

**FIG. 5:**
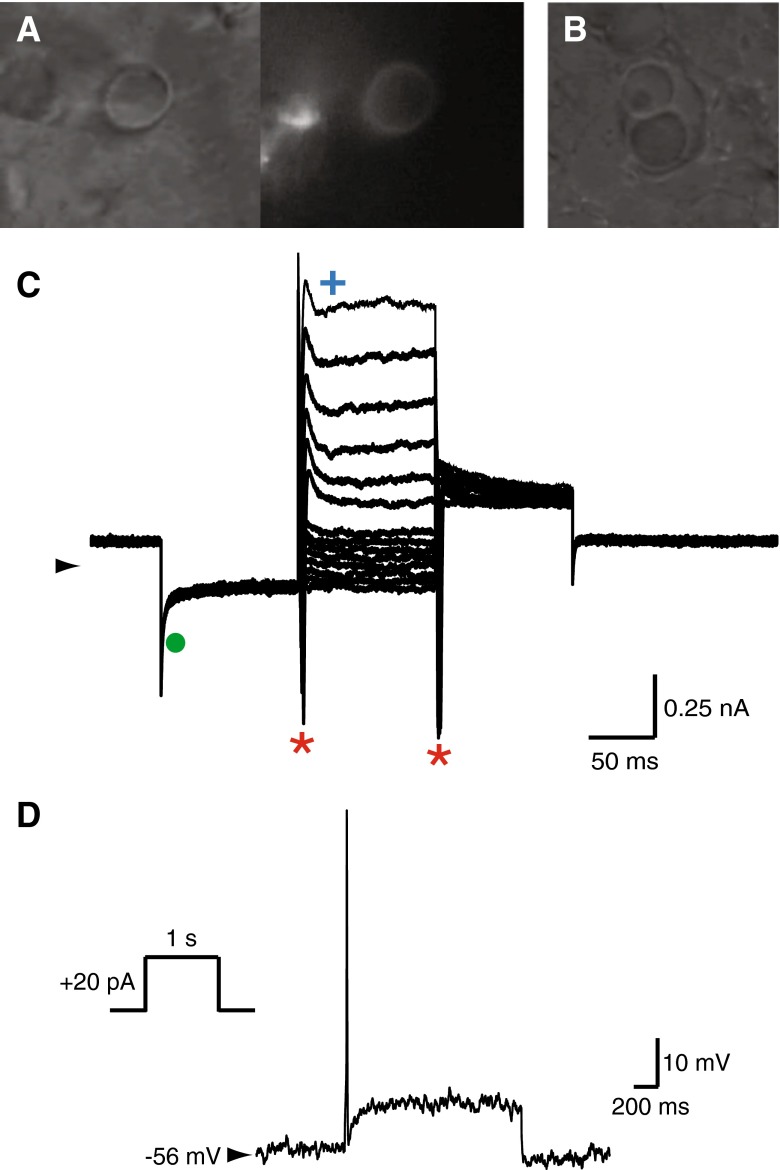
Recordings from a calyx primary afferent terminal in 15 WG fetal vestibular neuroepithelium. **A** Infrared DIC image shows a calyx terminal (*viewed from above*) that appears as a ring. Fluorescent image shows a similar “halo” after filling the terminal with Alexa-594 dye during recording. Note: hollow appearance of halo. **B** Complex calyces were also observed at this age. **C** Voltage clamp recording from the calyx primary afferent terminal shows activation of potential I_K,L_ (*filled green circle*), Na^+^ (*red asterisks*), and K^+^ (*blue cross*) whole-cell currents in response to voltage activation protocol (as shown in Fig. 2A inset). **D** Current clamp recording shows a single overshooting action potential generated by a depolarizing current step injection (inset) in the same calyx primary afferent terminal.

Anatomical studies have shown that calyceal primary terminals begin to envelop presumptive type I hair cells in central regions of human cristae and maculae as early as ∼9 WG (Sans et al. 1994). However, we could not obtain recordings from calyceal terminals younger than 15 WG. Using IR-DIC imaging, we observed a ring-like structure of a calyx terminal in the human crista similar to those described in mouse (Fig. 5A left, see also Fig. 4, Eatock and Songer 2011). Subsequent imaging of intracellular Alexa-594 fluorophore confirmed a calyceal “halo” characteristic (Fig. 5A, middle). This halo is markedly different to the solid-filled hair cell shown in Figure 3A. Recordings from the same calyx show inward and outward currents that are presumably due to Na^+^ and K^+^ channels respectively in this highly specialized afferent terminal. Inward currents (Fig. 5B, asterisks) are evident in response to depolarizing current steps from hyperpolarized membrane potentials. In rodents, these have been identified as voltage activated Na^+^ currents, typical of calyx terminals, and are blocked by TTX (Dhawan et al. 2010). The identity of this current has yet to be confirmed in human calyces. In addition, there appears to be more than one whole-cell K^+^ conductance in calyx terminal recordings (Fig. 5B). Upon hyperpolarization to −129 mV, a conductance that resembles *G*_K,L_ was observed (filled circle). At more depolarized potentials, the whole-cell currents have fast and slow activating and inactivating characteristics. Previous studies report that calyceal K^+^ currents not only have *G*_K,L_ but also TEA, 4-AP, linopirdine, and apamin sensitive components (Hurley et al. 2006; Rennie and Streeter 2006; Dhawan et al. 2010). Pharmacological characterization of all these outward currents in developing human calyces is yet to be completed. In older tissue (>15 WG), we have also observed what appear to be complex calyces (Fig. 5A, right). To date, we have not recorded from these presumed complex calyces.

In current clamp, the average resting membrane potential of developing human calyx terminals was −64 ± 4 mV (*n* = 3). Current clamp recordings from rodent calyceal terminals typically show that a single action potential (AP) is discharged during depolarization (Rennie and Streeter 2006; Dhawan et al. 2010). Similarly, the human calyx recording shown in Figure 5C, with a resting membrane potential of −56 mV, also discharged a single AP in response to a depolarizing current step of 20 pA (Fig. 5C). We could not elicit multiple AP’s with increasing level of current injection. The AP had a 10 mV overshoot and showed a small after hyperpolarization, similar to that observed in mature gerbil calyces (Meredith et al. 2011). Our data, therefore, indicate that calyx terminals are not only present in the developing neuroepithelium but may also be functionally active as early as 15 WG. We have recordings from three calyceal terminals from human neuroepithelium aged 15, 17, and 18 WG, although only the calyx aged 18 WG discharged action potentials. However, we did not see evidence of synaptic quantal events in any of our voltage clamp recordings from calyceal terminals. Thus, it is unclear if the calyx synapse is fully functional at this early stage of development.

## DISCUSSION

In this study, we established a semi-intact preparation of the human fetal vestibular organs and obtained the first whole-cell current recordings from developing human vestibular hair cells and the *only* recordings from human calyx primary afferent terminals. Our major finding is that the gestational period examined (11–18 WG) represents a crucial transitional phase where the mature functional characteristics of type I and type II hair cells emerge.

### Recordings from Hair Cells

From our data, 11 to 14 WG marks the end of a nascent phase where type II vestibular hair cells express whole-cell conductances similar to, albeit smaller than, more mature fetal human hair cells (15–18 WG). Our results indicate that there is a significant increase in *G*_MAX_ of type II hair cells during development. This increase in *G*_MAX_ was not associated with a change in *V*_½_ or *S*, the slope of the activation curve. This suggests the expression of a greater number of voltage-activated currents in type II hair cells with age rather than a change or an upregulation of different channel types. However, both fetal (this study) and adult (Oghalai et al. 1998) *G*_MAX_ values recorded in human hair cells were smaller than those in rodents (Rüsch et al. 1998). These reduced *G*_max_ values would have the effect of increasing hair cell input resistance. Therefore, for a given stimulus, the voltage gain would be enhanced, presumably resulting in more neurotransmitter release.

Increased input per human hair cell onto afferent terminals is amplified still further by the significantly increased convergence of human hair cells onto primary afferent terminals compared to rodents. A recent study estimated there were 36,000 hair cells in both fetal (16 WG) and adult human utricle (Severinsen et al. 2010). This is almost an order of magnitude greater (36,000 versus. 3,800) than values reported for the same structure in mice (Desai et al. 2005b). There are approximately 3,400 utricular afferent fibers in humans (Bergstrom 1973) and ∼680 in mice (Desai et al. 2005b) resulting in an increased ratio of hair cells per afferent (∼10:1 versus ∼5:1). A similar ratio also exists for cristae between human and mouse. In *adult* human cristae, the ratio of hair cells to afferent fibers is 5:1 (∼8,000 hair cells; ∼1,400 afferents; Lopez et al. 2005a; Lopez et al. 2005b), while in mouse cristae, the ratio is 1:2 (∼1,420 hair cells; ∼680 afferents; Desai et al. 2005a). Precisely, why there is potentially more hair cell transmitter release and greater convergence onto afferent terminals in humans than rodents is unclear, but these results suggest that human afferent discharge thresholds may be higher than those in rodents.

During the next phase of development (15–18 WG), there is continued maturation where adult-like features of the vestibular neuroepithelium begin to emerge. At this stage, whole-cell voltage-activated currents were more diverse and conductances were larger than earlier stages of development but still smaller than those observed in adult human hair cells (Oghalai et al. 1998). Indeed, 15 WG appears to be a milestone in hair cell development where an apparent small *G*_K,L_ begins to emerge, resulting in the functional segregation of two hair cell types. Similar to other outward conductances described above, initially, *G*_K,L_ is small and likely to increase in amplitude during development as has been observed in developing mouse vestibular hair cells (Geleoc et al. 2004). Our results also suggest that this physiological differentiation, as determined by the expression of *G*_K,L_, precedes morphological differentiation, as a recent study could not unambiguously distinguish type I and type II hair cells at 16 WG (Severinsen et al. 2010).

While future pharmacological studies will include antagonists such as linopirdine and XE-991 to establish the presence of *G*_K,L_ in putative type I hair cells, there are several other features that suggest the increasing expression of *G*_K,L_. For example, low input resistance, a direct consequence of *G*_K,L_ expression marks the physiological differentiation of type I hair cells. In mice, this lowered input resistance is first observed at E18 (Geleoc et al. 2004) and becomes more prevalent in both mice and rats during the first postnatal week (Rüsch et al. 1998; Geleoc et al. 2004). Our data show that putative human type I vestibular cells also exhibit significantly lower input resistances at ∼15 WG compared to type II hair cells. It has also been noted that *G*_K,L_ activates at more positive membrane potentials in neonatal rats (average *V*_½_ = 34 mV more depolarized in the first compared to the second postnatal week, Hurley et al. 2006). Our data are also consistent with these observations, as fetal *G*_K,L_ is activated at more depolarized potentials (∼−59 mV; see Fig. 4) than reported in adult human vestibular hair cells that have an activation range of approximately −90 mV (Oghalai et al. 1998). Rodent data also show that the magnitude of *G*_K,L_ in hair cells increases with age as does *G*_MAX_ in cultured and acutely isolated epithelial preparations (Rüsch et al. 1998). Due to the collapse of tail currents at potentials more depolarized than ∼−29 mV, we could not determine *G*_MAX_ in fetal human type I hair cells. As mentioned above, collapse of the activation curve is only observed in preparations that maintain the cellular microarchitecture around type I hair cells (Lim et al. 2011) and is consistent with the presence of partial or complete calyceal primary afferents surrounding presumed type I hair cells (see Fig. 4C). The presence of small but distinct *G*_K,L_ as well as collapsing tail currents supports the notion that physiological differentiation of type I hair cells occurs at approximately 15 WG in human fetal hair cells.

Our data also shows that there is a transient expression of Na^+^ channels in human hair cells up to 14 WG. In rodent hair cells, Na^+^ conductances have markedly differing cellular, regional, and developmental expression profiles (Rüsch et al. 1998; Geleoc et al. 2004; Wooltorton et al. 2007; Li et al. 2010). Two types of Na^+^ currents have been identified in rats; I_na,1_ is found in all type I hair cells throughout development, whereas I_Na,2_, was present only in subsets of hair cells until P7 (Wooltorton et al. 2007). Our data show that *V*_1/2 act_ and *V*_1/2 deact_ values for presumptive Na^+^ currents in human hair cells are similar to those described for I_na,1_ in developing rat hair cells (Wooltorton et al. 2007). Pharmacological characterization of presumptive Na^+^ currents in fetal human hair cells is required to determine which subtypes are expressed during the developmental period examined.

The transient expression of Na^+^ channels during development is thought to result in AP generation and maybe important for release of trophic factors necessary for synaptic maturation (Chabbert et al. 2003). We observed Na^+^ conductances in hair cells over a period where the anatomical substrates of synapse formation appear (Sans et al. 1994) but prior to clear morphological differentiation of type I hair cells. Taken together, our data suggests the decline in Na^+^ conductances from human hair cells coincides with the increased expression of *G*_K,L_ and the time when definitive calyceal recordings are first obtained.

### Calyx Observations

Our data suggests a discrepancy between anatomical and physiological maturation of vestibular primary afferent terminals. Morphological studies show that penetration of the human vestibular neuroepithelium by primary afferents begins at ∼6.5 WG (Yokoh 1974), whereas contact with hair cells occurs by 9 WG (Sans and Dechesne 1985). Synaptic specializations including pre- and postsynaptic densities are also evident at this developmental stage (Sans and Dechesne 1985). This advanced development of synaptic elements suggests that calyceal terminals maybe functional very early during human development. The physiological readout of a “functional synapse” would be the presence of quantal synaptic events, appearing as brief miniature postsynaptic currents in calyceal voltage clamp recordings. This would indicate the activation of calyx postsynaptic receptors by neurotransmitter release from hair cells. Since we did not observe any quantal events in our calyceal recordings, this suggests three possibilities: (1) the synapses may not be mature (Songer and Eatock 2013); (2) damaged or immature hair bundles and consequently transduction apparatus may result in hyperpolarized hair cell membranes thereby reducing transmitter release; or (3) quantal release was compromised in our preparations. Although our current experimental setup could not distinguish between these three possibilities, it should be noted that calyx afferent terminals possess whole-cell conductances necessary for AP discharge by 18 WG. This would suggest that if the calyx/hair cell synapses were functional, then these signals could be transmitted by the afferent fiber to the CNS. Nevertheless, it still remains to be determined exactly when the human hair cell/calyx synapse becomes functionally active.

In short, while morphological data suggest that development of the unique hair cell/calyx synapse is around 8–9 WG, this appears to be well in advance of physiological maturation of *G*_K,L_ in type I hair cells and the AP-generating conductances in afferents that have emerged by ∼15 WG. The chronology of afferent innervation and hair cell physiological differentiation in humans is therefore consistent with findings in rats where the encapsulation of presumed type I hair cells by calyces occurs independently and in advance of hair cell differentiation and the acquisition of *G*_K,L_ (Rüsch et al. 1998). It should be noted that although *G*_K,L_ is typically associated with type I vestibular hair cells, it has also been described in rat and gerbil calyceal terminals (Hurley et al. 2006; Rennie and Streeter 2006; Dhawan et al. 2010). Our recordings from human calyx terminals provide similar evidence for nascent *G*_K,L_.

## CONCLUSIONS

Anatomical evidence suggests that the human vestibular neuroepithelium is well developed by 14 WG (Rosenhall 1972); however, prior to our study, functional data were not available. Although we know that hair cell innervation begins early in fetal development (9WG, Sans et al. 1994; Sans and Scarfone 1996), our data suggests that at this stage of development, hair cell conductances are not mature compared to adult human hair cells (Oghalai et al. 1998). By 15 WG, however, we observed early signs of *G*_K,L_ in some hair cells. The presence of hair-cell-specific conductances, together with AP generation in calyx terminals, (as well as prior studies on afferent myelination at 8–9 WG, Sanchez-Fernandez and Rivera-Pomar 1983) implies that the vestibular neuroepithelium has the necessary machinery to transmit sensory signals to the CNS early in the second trimester of pregnancy. This prediction would be dependent on the mechanosensory transduction channels (associated with the hair bundles of hair cells) being functionally operational. While this has yet to be confirmed in human tissue, it appears to be a reasonable assumption since transduction signals can be evoked early in mouse vestibular and auditory hair cell development (E17–P0; Geleoc and Holt 2003; Lelli et al. 2009).

Understanding the timing of morphological and physiological development of human hair cells and their primary afferent terminals is critical if we are to apply regenerative technologies to human inner ears. Studies have shown that the processes involved in regeneration and recovery from injury often recapitulate those observed during development (Levic et al. 2007). Given the precise spatiotemporal patterning of ion channel expression and coincident primary afferent innervation, future human experiments will need to target the concomitant molecular signals that are necessary for cell survival, specification, differentiation, ion channel expression, and synaptogenesis. Some of these signals, whose appearance is fleeting during the compressed embryogenesis in rodents, presumably persist longer in the extended gestational period of humans. We propose that our semi-intact preparation presents a new opportunity for combined functional, anatomical, and molecular investigation of fetal hair cell development in humans.
